# Device-measured sedentary behavior in oldest old adults: A systematic review and meta-analysis

**DOI:** 10.1016/j.pmedr.2021.101405

**Published:** 2021-05-18

**Authors:** Katelyn E. Webster, Weijiao Zhou, Nancy A. Gallagher, Ellen M. Lavoie Smith, Neha P. Gothe, Robert Ploutz-Snyder, Natalie Colabianchi, Janet L. Larson

**Affiliations:** a University of Michigan School of Nursing, 400 North Ingalls Building, Ann Arbor, MI 48109, USA; b University of Illinois at Urbana-Champaign College of Applied Health Sciences, 1206 South Fourth St., Champaign, IL 61820, USA; c University of Michigan School of Kinesiology, 1402 Washington Heights, Ann Arbor, MI 48109, USA

**Keywords:** Older adults, Physical activity, Sitting, Accelerometry, activPAL

## Abstract

Sedentary behavior contributes to health decline and frailty in older adults, especially the oldest old. The purpose of this systematic review was to synthesize evidence describing the volume of device-measured sedentary behavior and factors that influence sedentary behavior in community-dwelling adults aged 80 and older. Four electronic databases were searched in August 2018; the search was updated in September 2019 and December 2020. Twenty-one articles representing 16 unique datasets from six countries met inclusion criteria. Various devices and data processing methods were used to measure sedentary behavior; the most common device was the ActiGraph accelerometer. Sedentary time during the waking day ranged from 7.6 to 13.4 h/day. Studies using similar measurement methods (hip-worn ActiGraph with uniaxial cut-point <100 counts per minute) had a weighted mean of 10.6 h/day. Subgroup analyses revealed that male gender and age ≥85 may contribute to increased sedentary behavior. Only seven individual articles examined factors that influence sedentary behavior in the 80 and older age group; older age, male gender, non-Hispanic white race/ethnicity, social disadvantage, and declining cognitive function (in men) were associated with increased sedentary behavior. In conclusion, the oldest old are highly sedentary and little is known about factors that influence their sedentary behavior.

## Highlights

• This literature review found that adults ≥ 80 years are sedentary 10.6 h/day.
• Five personal factors were associated with sedentary behavior in adults ≥ 80 years.
• Factors influencing sedentary behavior in adults ≥ 80 years are understudied.

## 1 Introduction

Older adults are the most sedentary age group in the United States (Matthews et al., 2008). Growing evidence suggests that sedentary behavior (SB) contributes to health decline and frailty in older adults, especially the oldest old (≥80-85 years) (Leitzmann et al., 2018, Dogra and Stathokostas, 2012, Valenzuela et al., 2019). SB is defined as any behavior with a low energy expenditure (≤1.5 metabolic equivalents) in a sitting, reclining, or lying position while awake (Tremblay et al., 2017). Increased SB has been associated with lower odds of successful aging in physical, psychological, and sociological domains; (Dogra and Stathokostas, 2012) increased risk of developing physical frailty; (Song et al., 2015) and increased risk of disability in activities of daily living and instrumental activities of daily living (Chen et al., 2016, Dunlop et al., 2015). Although the *Physical Activity Guidelines for Americans* now suggest that older adults replace sedentary time with light physical activity (Department of Health and Human Services, 2018), no guidelines currently exist on limiting SB to a specific number of hours per day.

While most SB literature has been based on self-reported sedentary time, evidence based on device-measured SB is increasing (Leitzmann et al., 2018). Because SB is typically not a planned activity and takes place in the context of everyday life, recalling daily volumes of sedentary time is difficult and self-report measures are often biased (Gennuso et al., 2015). Devices such as accelerometers and inclinometers are precise, can be used to measure SB objectively, and are more accurate than self-report (Kozey-Keadle et al., 2011).

Previous reviews found that older adults, aged ≥60 or 65, are sedentary; device-measured SB ranged from 8.5 to 9.6 h/day (Harvey et al., 2015, Wullems et al., 2016). One review that synthesized literature on determinants of SB in adults 65 and older found evidence for personal (age, retirement, obesity, health status), interpersonal (loneliness/living alone), and environmental (mode of transportation, housing type, neighborhood characteristics) factors (Chastin et al., 2015). However, since functional fitness and physical capability generally decrease with age (Milanović et al., 2013, Cooper et al., 2011), SB patterns and influencing factors may be different for the younger old (60 or 65–80 years) compared to the oldest old (≥80-85 years).

While much evidence for SB in older adults does exist, fewer studies focus on those ≥80 years. Because these individuals are at higher risk for health decline and frailty and this population is growing as people live longer (Zhang et al., 2020, Barnett et al., 2012, Lee et al., 2020), a review focused on the volume of SB and the factors that influence SB in the oldest old population is needed. Synthesizing the existing evidence about the volume of SB in this age group will be helpful as more is known about what thresholds of sedentary time are associated with harmful health effects (Ku et al., 2018). Also, understanding factors that influence SB may guide the development of interventions to reduce SB; this could promote aging in place by retention of functional abilities, prevent or treat frailty, improve quality of life into later years, and reduce healthcare utilization and costs associated with low physical function and frailty (Lerma et al., 2018, Copeland et al., 2017, Kim and Lee, 2019, Hoogendijk et al., 2019, Cheng et al., 2020). This systematic review of the literature aims to characterize the volume of device-measured SB and to identify factors that may influence SB in community-dwelling adults aged 80 and older.

## 2 Methods

This review was conducted according to systematic review guidelines by Siddaway et al. (2019) and reported according to guidelines from the Preferred Reporting Items for Systematic Reviews and Meta-Analyses (PRISMA) (Moher et al., 2009). In consultation with a health sciences informationist, a search was conducted in four databases: PubMed, CINAHL, AgeLine, and Scopus. Search terms were related to older adults, SB, and devices that measure SB. Subject headings and indexed terms were included as appropriate for each database (see full search strategies in Box 1). The search was initially conducted in August 2018 and updated using the same methods in September 2019 and December 2020. The search was not restricted by year of publication.

Box 1 **Search strategies by database.**

AgeLine

(DE “80+” OR DE “85+” OR DE “90+” OR DE “95+” OR DE “Centenarians” OR DE “Old Old” OR DE “Older Adults” OR DE “Young Old” OR (elderly OR “senior citizen” OR geriatric OR “older adult”)) AND (Accelerometry OR accelerometer OR accelerometers OR Actigraphy OR actigraph OR actigraphs OR activpal OR Actical OR sensecam OR inclinometer OR inclinometers OR inclinometric OR inclinometry) AND ((DE “Sedentary Lifestyle”) OR (Sedentary OR inactivity OR inactive))

CINAHL

((MH “Geriatrics”) OR (MH “Aged”) OR (MH “Aged, 80 and Over”) OR (elderly OR “senior citizen” OR geriatric OR “older adult”)) AND ((MH “Accelerometers”) OR (MH “Accelerometry”) OR (MH “Actigraphy”) OR (Accelerometry OR accelerometer OR accelerometers OR Actigraphy OR actigraph OR actigraphs OR activpal OR Actical OR sensecam OR inclinometer OR inclinometers OR inclinometric OR inclinometry)) AND ((MH “Life Style, Sedentary”) OR (Sedentary OR inactivity OR inactive))

PubMed

(“Sedentary Behavior”[Mesh] OR “Sitting Position”[Mesh] OR “Sedentary Lifestyle”[Mesh] OR Sedentary[tw] OR inactivity[tw] OR inactive[tw]) AND (“Accelerometry”[Mesh] OR Accelerometry[tw] OR accelerometer[tw] OR accelerometers[tw] OR “Actigraphy”[Mesh] OR Actigraphy[tw] OR actigraph[tw] OR actigraphs[tw] OR activpal[tw] OR Actical[tw] OR sensecam[tw] OR inclinometer[tw] OR inclinometers[tw] OR inclinometric[tw] OR inclinometry[tw]) AND (“Geriatrics”[Mesh] OR “Aged, 80 and over”[Mesh] OR “Aged”[Mesh] OR elderly[tw] OR “senior citizen”[tw] OR geriatric[tw] OR “older adult”[tw]))

Scopus

TITLE-ABS-KEY((elderly OR “senior citizen” OR geriatric OR “older adult”) AND (Accelerometry OR accelerometer OR accelerometers OR Actigraphy OR actigraph OR actigraphs OR activpal OR Actical OR sensecam OR inclinometer OR inclinometers OR inclinometric OR inclinometry) AND (Sedentary OR inactivity OR inactive))

Articles included in this systematic review met all of the following criteria: used observational/population-based research design, included participants who were community dwelling and aged 80 years and older, reported device-measured sedentary time or percentage of the day spent sedentary, were conducted in a free-living environment, and were written in English. Studies were excluded if they provided less than three days of activity monitoring. To avoid biasing the results of this review, studies were excluded if their recruitment targeted subjects with a specific activity level or a specific condition or disease (e.g., osteoarthritis, obesity, or diabetes). Studies were not automatically excluded if they included subjects younger than 80 years; however they needed to report a sedentary time specific to subjects ≥80 years.

**Fig. 1:**
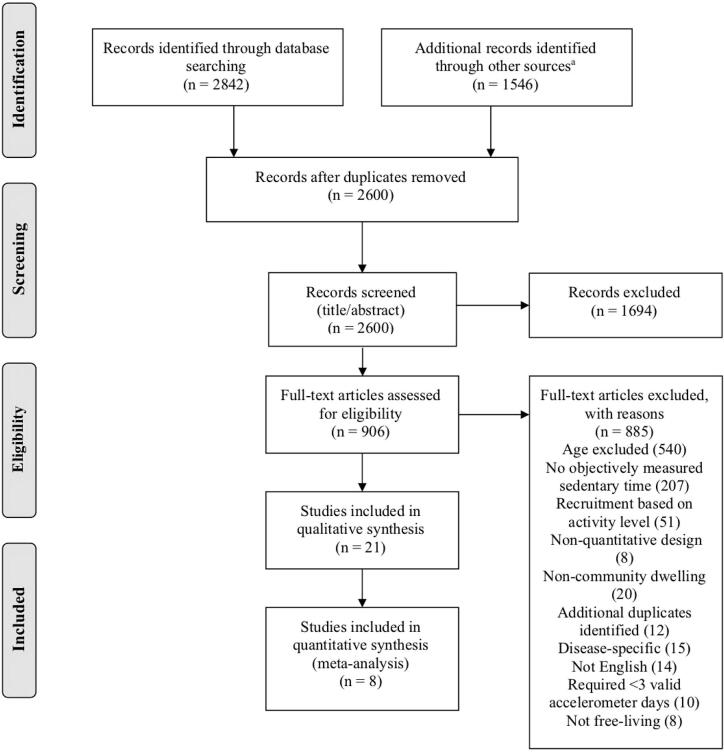
PRISMA flow diagram. ^a^ Other sources included reference lists of included articles, articles that have cited included articles, and grey literature.

After removing duplicate articles, two independent reviewers screened each title/abstract using a checklist of the inclusion and exclusion criteria. The full texts of studies that appeared to meet criteria were then reviewed, and any discrepancies in the findings of the two reviewers were resolved in meetings. Additional searching for qualifying studies included checking the reference lists of included articles, using Scopus to find articles that cited the included studies, and locating any qualifying grey literature using Google searches for keywords *sedentary behavior* and *older adults* on the websites of relevant professional organizations (American College of Sports Medicine, Society of Behavioral Medicine, Sedentary Behavior Research Network). These additional articles were reviewed via the same process used for those retrieved from the databases. Articles meeting all inclusion and exclusion criteria were included in the systematic review. A PRISMA diagram outlining the literature review process is shown in Fig. 1.

Two independent reviewers extracted the following data from each article: the country where the study was conducted, sample size, study design, devices used to measure SB, device wear location, minimum number of valid monitoring days, non-wear algorithm used (if applicable), uniaxial or triaxial data used (if applicable), cut-points used to determine SB or definition of SB used, when subjects were asked to wear the device (waking hours vs. 24 h/day), how sleep time was addressed with 24-hour data, mean wear time, sedentary time reported (with measure of variability), and factors that influenced SB or were associated with SB. Some studies reported separate estimates of sedentary time by gender or narrower age categories, which we also extracted. Sedentary times reported as minutes/day were converted to hours/day and measures of variability were converted to standard errors and 95% confidence intervals. We attempted to contact authors of studies that were potentially eligible for the meta-analysis, but were missing the sample size for subjects ≥80. Two reviewers conducted a quality assessment of each article using the Quality Assessment Tool for Observational Cohort and Cross-Sectional Studies and rated each article as *good*, *fair*, or *poor* based on 14 criteria such as participation rate; application of inclusion/exclusion criteria; sample size justification; timing, validity, and reliability of exposure and outcome measures; loss to follow-up; and adjustment for confounding variables (National Heart, 2014).

For studies that used similar measurement methods, sedentary time results were quantitatively synthesized to report an overall mean sedentary time weighted by the inverse standard error with a fixed effects model. We also conducted subgroup analyses to compare mean sedentary times by gender and age (younger vs. older) subcategories. Forest plots were created with weighted mean sedentary times and the I^2^ statistic was used to assess heterogeneity. These analyses were conducted with Stata 15.1 software (Stata Statistical Software, 2017). Measurement approaches (devices, cut-points, non-wear algorithms) and any factors associated with SB were summarized.

## 3 Results

Of the 2,600 non-duplicate articles retrieved from the four databases and additional searches, a total of 21 met all criteria for inclusion (see Table 1). Articles were published from 2011 to 2020 and represented 16 unique datasets from studies conducted in six countries (Iceland, Japan, Norway, Portugal, United Kingdom, United States). Three of the datasets were nationally representative samples from Norway (Sagelv et al., 2019), Portugal (Santos et al., 2018), and the United States (two waves of the National Health and Nutrition Examination Survey) (Dunlop et al., 2015, Chastin et al., 2014, Evenson et al., 2012, Evenson et al., 2014). Twelve articles utilized data from large cohort studies (Sagelv et al., 2019, Arnardottir et al., 2013, Berkemeyer et al., 2016, Chen et al., 2015, Çukić et al., 2018, Hooker et al., 2016, Jefferis et al., 2015, Okely et al., 2019, Shaw et al., 2017aa, Shaw et al., 2017bb, Rosenberg et al., 2020, Suzuki et al., 2020).

**Table 1:** Data extracted from included studies for subjects 80 years and older.

Article Author (Year Published)	Study Name, Country, Study design	Device Used, Cut-Point or Determination of SB, Uniaxial or Triaxial Data Used (if relevant)	Device Wear Location	Gender (if reported separately), Age Group (years)	Sample Size	Mean Sedentary Time per Day During Waking Hours^a^	95% Confidence Interval (or Interquartile Range)	Variables Analyzed for Association with Sedentary Behavior (only significant if noted)	Quality Assessment Rating
Arnardottir et al. (2013)^b^	AGESII-Reykjavik, Iceland, Cross-sectional	ActiGraph GT3X <100 cpm Uniaxial	Right hip	Women 80–84	90	10.0	9.7–10.3	–	Fair
				Women ≥85	65	10.2	9.9–10.5		
				Men 80–84	64	10.7	10.4–11.0		
				Men ≥85	28	10.7	10.1–11.3		
Berkemeyer et al. (2016)	EPIC- Norfolk, UK, Cross-sectional	ActiGraph GT1M <100 cpm Uniaxial	Right hip	Women >80	165^c^	10.2 (median)	9.4, 11.2 (IQR)	–	Fair
				Men >80	205^c^	10.7 (median)	9.7, 11.6 (IQR)		
Chastin et al. (2014)	NHANES 2005-2006, US, Cross-sectional	ActiGraph AM7164 <100 cpm Uniaxial	Hip	Women ≥80	–	71.1%	–	–	Fair
				Men ≥80	–	72.5%	–		
Chen et al. (2015)	Sasaguri Genkimon, Japan, Cross-sectional	Active Style Pro HJA-350IT ≤1.5 METs Triaxial	Waist	Women ≥80	198	7.8	7.5–8.1	–	Good
				Men ≥80	108	8.6	8.2–9.0		
Çukić et al. (2018)	Seniors USP Twenty-07 1930s cohort, Scotland, Longitudinal^d^	activPAL3c Thigh position	Domin-ant thigh	Women mean age 83^e^	65	68.5%	66.0–70.9%	Gender, cognitive ability	Good
				Men mean age 83^e^	54	68.0%	64.8–71.1%		
Davis et al. (2011)^b^	Project OPAL, UK, Cross-sectional	ActiGraph GT1M <100 cpm Uniaxial	Waist	80–84	59	11.0	10.6–11.4	Age^f^	Fair
				≥85	28	12.2	11.6–12.8		
Dunlop et al. (2015)	NHANES 2003-2004 and 2005-2006, US, Cross-sectional	ActiGraph AM7164 <100 cpm Uniaxial	Waist	≥80	494	9.6	9.4–9.8	–	Fair
Evenson et al. (2012)	NHANES 2003-2004 and 2005-2006, US, Cross-sectional	ActiGraph AM7164 <100 cpm Uniaxial	Right hip	Women ≥80	305	8.9	8.6–9.1	Gender^f^, race/ethnicity^f^	Fair
				Men ≥80	278	9.4	9.1–9.6		
Evenson et al. (2014)^b^	NHANES 2003-2004 and 2005-2006, US, Cross-sectional	ActiGraph AM7164 <100 cpm Uniaxial	Right hip	≥80	555	10.2	10.0–10.4	–	Fair
(Evenson et al. 2014 continued)^b^	Cardiovas-cular Health of Seniors and the Built Environment, US, Cross-sectional	ActiGraph GT1M and GT3X <100 cpm Uniaxial		≥80	155	10.6	10.2–11.0		
Hooker et al. (2016)	REGARDS, US, Cross-sectional	Actical <50 cpm	Right hip	≥85	–	13.4	13.2–13.5	–	Fair
Jefferis et al. (2015)^b^	British Regional Heart, UK, Cross-sectional	ActiGraph GT3X <100 cpm Uniaxial	Hip	Men ≥80	470	10.7	10.6–10.8	–	Fair
Lohne-Seiler et al. (2014)^b^	Unnamed, Norway, Cross-sectional	ActiGraph GT1M <100 cpm Uniaxial	Right hip	Women 80–85	37	9.9	9.5–10.3	–	Fair
				Men 80–85	28	9.8	9.4–10.2		
Okely et al. (2019)	Seniors USP Twenty-07 1930s cohort, Scotland, Longitudinal^d^	activPAL3c Thigh position	Domin-ant thigh	Mean age 83^e^	118	68.2%	66.2-70.1%	Depression, anxiety	Good
Rosenberg et al. (2020)^g^	Adult Changes in Thought, US, Cross-sectional	activPAL micro Thigh position	Thigh	80–84	173	10.1^h^	9.8–10.5	–	Fair
				85–89	114	10.4^h^	10.0–10.8		
				≥90	49	11.6^h^	11.1–12.1		
		ActiGraph wGT3X+ ≤18 vector magnitude counts/15 seconds Triaxial	Right supra-iliac crest	80–84	185	9.5	9.2–9.7		
				85-89	112	10.1	9.8–10.4		
				≥90	51	10.5	10.1–10.8		
Ryan et al. (2019)	Unnamed, UK, Cross-sectional	GENEActiv Original Seated/reclined position with <0.057 Residual G (<1.5 METs) Triaxial	Domin-ant thigh	≥84	9	10.5	9.7–11.2	–	Fair
Sagelv et al. (2019)^b^	Tromsø, Norway, Cross-sectional	ActiGraph wGT3X-BT <100 cpm Uniaxial	Right hip	≥80	235	11.6	11.4–11.8	–	Fair
		<150 vector magnitude cpm Triaxial				9.6	9.3–9.8		
Santos et al. (2018)^b^	Unnamed, Portugal, Cross-sectional	ActiGraph GT1M <100 cpm Uniaxial	Right hip	Women 80–84	44	9.5	8.8–10.2	–	Fair
				Women ≥85	47	10.2	9.7–10.7		
				Men 80–84	38	9.4	8.8–10.0		
				Men ≥85	27	9.6	8.8–10.4		
Shaw, Cukic, Deary, Gale, Chastin, Dall, Dontje et al. (2017)	Seniors USP Twenty-07 1930s cohort, Scotland, Longitudinal^d^	activPAL3c Thigh position	Domin-ant thigh	Mean age 83^e^	119	68.2%	66.2–70.2%	Neighborhood environment, social participation, social support, and home environment	Fair
Shaw, Cukic, Deary, Gale, Chastin, Dall, Skelton et al. (2017)	Seniors USP Twenty-07 1930s cohort, Scotland, Longitudinal^d^	activPAL3c Thigh position	Domin-ant thigh	Mean age 83^e^	119	68.2%	66.2–70.2%	Multiple measures of socioeconomic position^f^	Fair
Suzuki et al. (2020)^i^	Arakawa 85+, Japan, Cross-sectional	ActiGraph GT3X <100 cpm^j^	Waist	Women mean age 88	68	13.3^k^	12.7–13.9	Gender^f^, cognitive function^l^	Fair
				Men mean age 88	68	14.2^k^	13.7–14.8		
Yonemoto et al. (2019)	Hisayama, Japan, Longitudinal	Active Style Pro HJA-350IT ≤1.5 METs Triaxial	Waist	≥80	23	7.6 (median, measured in 2009)	7.0–9.8 (IQR)	–	Fair
						8.9 (median, measured in 2012)	7.2–10.4 (IQR)		

Abbreviations: cpm, counts per minute; IQR, interquartile range; METs, metabolic equivalents; SB, sedentary behavior; UK, United Kingdom; US, United States. A hyphen indicates sample size was not reported for this age group, 95% CI was not reported and could not be calculated, or the study did not analyze any factors associated with sedentary behavior in subjects age ≥80.

a Hours/day or % of day in sedentary behavior (Mean unless otherwise noted as median).

b Unique studies included in meta-analysis.

c Sample size was not reported in this article (and authors did not respond to a request for information), but was found in another article about the study sample (Wu et al., 2017).

d Predictors of sedentary behavior were measured in earlier waves of the study and sedentary behavior was measured in a later wave.

e Note: the age range of this cohort was not totally clear, but subjects were born around 1932 (Shaw et al., 2017) with a mean age of 83.4 (SD 0.62) strongly indicating they meet criteria for this review ^.^

f Factor was significantly associated with sedentary behavior.

g This study excluded subjects in nursing homes, but it is not known if any subjects resided in other types of residential living.

h Specifically sitting time (rather than sitting and lying).

i Authors labeled this study community-dwelling, but we noted that one female subject resided in a nursing home.

j This study did not specify if uniaxial or triaxial data were analyzed.

k This study did not exclude sleeping time.

l Factor was significantly associated with sedentary behavior in men only.

According to the quality assessment, three included studies were rated as *good* and the other 18 were *fair*. The most common risks of bias were related to the cross-sectional design of 16 studies because sedentary time was measured at the same time point as any potential influencing factors. Other risks of bias were related to participation rates <20% and lack of sample size justifications.

The majority of studies used various ActiGraph accelerometer models to measure SB (Dunlop et al., 2015, Jefferis et al., 2015, Sagelv et al., 2019, Santos et al., 2018, Chastin et al., 2014, Evenson et al., 2012, Evenson et al., 2014, Arnardottir et al., 2013, Berkemeyer et al., 2016, Rosenberg et al., 2020, Suzuki et al., 2020, Davis et al., 2011, Lohne-Seiler et al., 2014). Two studies used Active Style Pro (Chen et al., 2015, Yonemoto et al., 2019) and one study each used Actical (Hooker et al., 2016) and GENEActiv (Ryan et al., 2019) accelerometers. Five articles representing two datasets utilized the activPAL device (four articles reported results from the same sample of subjects) (Çukić et al., 2018, Okely et al., 2019, Shaw et al., 2017aa, Shaw et al., 2017bb, Rosenberg et al., 2020). Accelerometer devices were mainly worn at the hip/waist; in one study, the accelerometer was worn on the thigh (Ryan et al., 2019). ActivPAL devices were worn on the anterior thigh (Çukić et al., 2018, Okely et al., 2019, Shaw et al., 2017aa, Shaw et al., 2017bb, Rosenberg et al., 2020). Most ActiGraph studies used uniaxial (vertical) data and a cut-point of <100 counts per minute to define SB. Two ActiGraph studies used triaxial data with cut-points of ≤18 vector magnitude counts per 15 seconds (Rosenberg et al., 2020) and <150 vector magnitude counts per minute (Sagelv et al., 2019). Studies that used other accelerometers either used smaller cut-points (<50 counts per minute) (Hooker et al., 2016), or metabolic equivalents (≤1.5 METS) to define SB (Chen et al., 2015, Yonemoto et al., 2019, Ryan et al., 2019). In the articles that used activPAL, SB was determined by thigh position (sitting or lying). Most studies used a non-wear algorithm to define times when the device was likely removed; minimum lengths of time ranged from 20 to 150 min of little or no activity. Appendix Table 1 provides a further description of data processing methods and wear time.

Reported sedentary times ranged from 7.6 to 13.4 h during the waking day. One study reported means of 13.3 and 14.2 sedentary h/day (for women and men respectively), but did not exclude sleep time (Suzuki et al., 2020). Some articles used a percentage of the waking day to report sedentary time, which ranged from 68.0% to 72.5% (Chastin et al., 2014, Çukić et al., 2018, Okely et al., 2019, Shaw et al., 2017aa, Shaw et al., 2017bb). We will primarily focus on studies that reported sedentary hours or minutes/day.

**Fig. 2:**
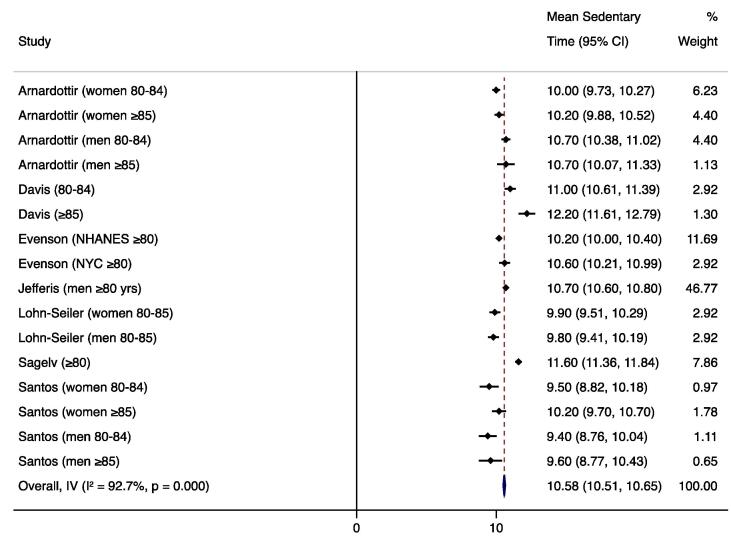
Forest plot of mean sedentary hours/waking day measured by hip-worn ActiGraph devices and processed using uniaxial data with a cut-point of <100 counts per minute.

**Fig. 3:**
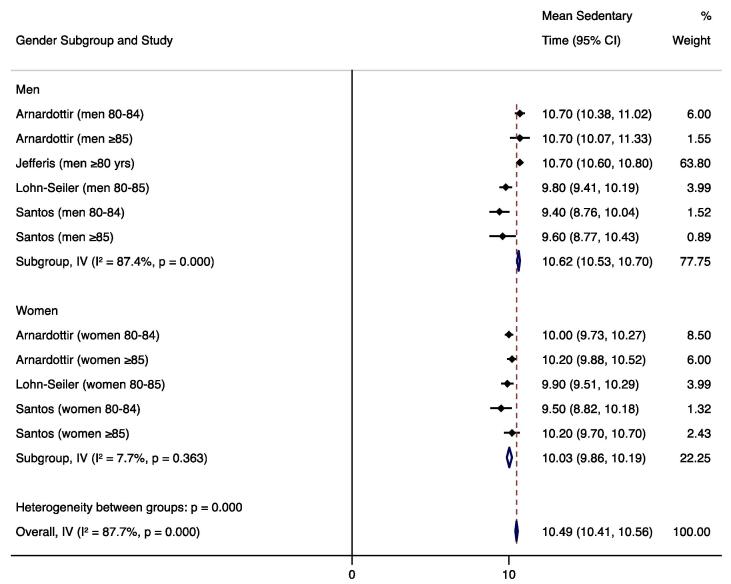
Forest plot of mean sedentary hours/waking day (measured by hip-worn ActiGraph devices and processed using uniaxial data with a cut-point of <100 counts per minute) by gender subgroups.

**Fig. 4:**
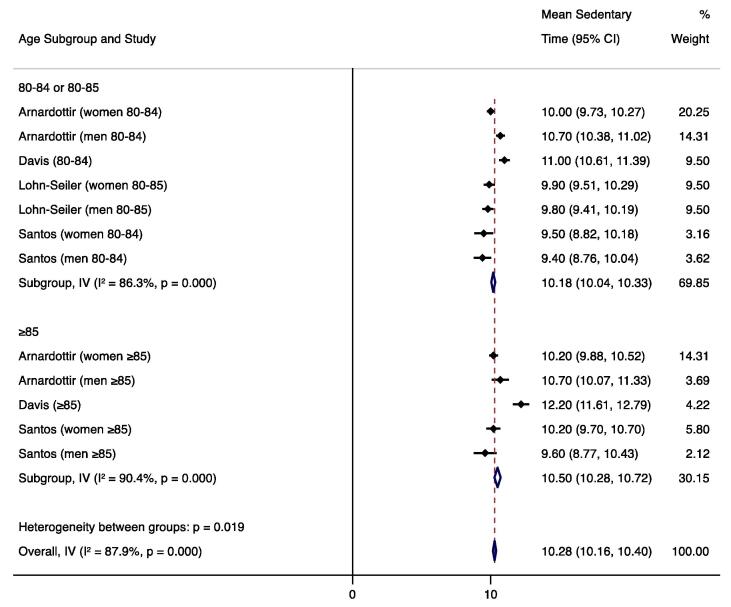
Forest plot of mean sedentary hours/waking day (measured by hip-worn ActiGraph devices and processed using uniaxial data with a cut-point of <100 counts per minute) by age subcategories.

We calculated the weighted mean sedentary time for studies that used hip/waist-worn ActiGraph uniaxial data with a SB cut-point of <100 counts per minute, the most common measurement method. We were not able to calculate weighted means of sedentary time for studies using alternate measurement methods because other devices and other ActiGraph cut-points were used in only one or two studies each. To avoid including duplicate subjects, only one NHANES estimate was included in the weighted mean; we included the study that required four (vs. three) valid days of monitoring and had the larger sample size (Evenson et al., 2014). One uniaxial ActiGraph study could not be included in the weighted mean because it reported the median sedentary time due to non-normal distribution (Berkemeyer et al., 2016). Mean sedentary estimates from seven articles (eight datasets) representing 1,970 total subjects were used to calculate the weighted mean (Sagelv et al., 2019, Santos et al., 2018, Evenson et al., 2014, Arnardottir et al., 2013, Jefferis et al., 2015, Davis et al., 2011, Lohne-Seiler et al., 2014), which was 10.6 h/day (95% CI 10.5, 10.7) during waking hours. Four studies included in the weighted mean reported results by gender; (Santos et al., 2018, Arnardottir et al., 2013, Jefferis et al., 2015, Lohne-Seiler et al., 2014) the subgroup analysis found significantly higher sedentary behavior in men (10.6 h/day; 95% CI 10.5, 10.7; n=655 total subjects) than in women (10.0 h/day; 95% CI 9.9, 10.2; n=283 subjects). Four studies included in the weighted mean reported results by narrower age categories; (Santos et al., 2018, Arnardottir et al., 2013, Davis et al., 2011, Lohne-Seiler et al., 2014) the subgroup analysis found significantly higher sedentary behavior in the ≥85 age group (10.5 h/day; 95% CI 10.3, 10.7; n=195 total subjects) than in those 80–84 or 80–85 (10.2 h/day; 95% CI 10.0, 10.3; n=360 subjects). Forest plots and I^2^ values are displayed in Fig. 2, Fig. 3, Fig. 4.

Studies that used activPAL, GENEActiv, and ActiGraph triaxial data reported sedentary estimates fairly similar to the uniaxial ActiGraph weighted mean (9.5–11.6 h/day) (Sagelv et al., 2019, Rosenberg et al., 2020, Ryan et al., 2019). The two studies that used the Active Style Pro device reported lower sedentary estimates (7.6–8.9 h/day) (Chen et al., 2015, Yonemoto et al., 2019) and the study that used Actical reported higher sedentary time (13.4 h/day) (Hooker et al., 2016).

Only seven articles examined factors associated with SB in the ≥80 age group; (Çukić et al., 2018, Davis et al., 2011, Evenson et al., 2012, Okely et al., 2019, Shaw et al., 2017bb, Shaw et al., 2017aa, Suzuki et al., 2020) few of these factors were shown to be significant. In one study, older age was associated with increased SB: participants age 85 and older were more sedentary than participants age 80–84 (Davis et al., 2011). In two studies, sedentary time differed by gender, with men being more sedentary than women; (Evenson et al., 2012, Suzuki et al., 2020) however, another study did not find gender differences (Çukić et al., 2018). For race/ethnicity, non-Hispanic whites had higher SB than did Hispanics (Evenson et al., 2012). Greater social disadvantage was associated with increased SB according to measures of residential area deprivation, social class, and car ownership (Shaw et al., 2017). One cross-sectional study found that declining cognitive function was associated with increased SB in men (Suzuki et al., 2020), but a longitudinal study found that cognitive ability did not predict SB (Çukić et al., 2018). Additional variables found to be non-significant in their association with SB in this age group included depression, anxiety, and neighborhood and social environment (Okely et al., 2019, Shaw et al., 2017aa). Although studies reported other factors associated with SB in older adults, they were not specific to those ≥80 years.

## 4 Discussion

This review revealed that adults aged 80 and older are sedentary for an average of 10.6 h during the waking day, as measured by ActiGraph uniaxial methods. Few of the reviewed studies evaluated factors that might influence SB in this age group; however, five factors were shown to be associated with increased SB—older age, male gender, non-Hispanic white race/ethnicity, social disadvantage, and declining cognitive function in men.

The mean device-measured sedentary time in the oldest old (≥80) was approximately one to two hours/day greater than that found in two previous reviews of adults aged 60 and older, which reported 9.4 and 8.5–9.6 h of device-measured SB during the waking day (Harvey et al., 2015, Wullems et al., 2016). This increase in SB with age is consistent with previous research of middle-aged and older adults where physical activity declined with age (Diaz et al., 2016, Schrack et al., 2014). Taken together, the evidence suggests that this is a robust and well documented relationship.

Our meta-analytic comparisons of mean sedentary time by gender revealed significantly higher sedentary behavior in men across four studies; however, these gender differences may be due to the high heterogeneity among studies (I^2^=87.7% overall). Similarly, the comparisons of narrower age categories revealed higher sedentary behavior in subjects ≥85 years compared to those 80–84 or 80–85, but we again observed high heterogeneity among studies (I^2^=87.9% overall). While these meta-analytic results were consistent with the results of the individual included studies, further research is needed to establish the effects of gender and age on SB within the oldest old population.

Cultural differences may influence SB in various countries (Koyanagi et al., 2018). The review did not include results from enough countries to draw conclusions, but it is interesting to note that the lowest estimates of sedentary time for men and women combined were from studies conducted in Japan (7.6 h/day and 8.1 h/day) (Chen et al., 2015, Yonemoto et al., 2019) and the highest was from a study conducted in the United States (13.4 h/day) (Hooker et al., 2016). This is consistent with World Health Organization data showing that adults in the United States are less active than adults in Japan (Organization, 2016). However, differences in the devices used, data processing methods, and sample age (≥80 vs. ≥85 years) may have also contributed to the large difference in sedentary time between these studies. Because the triaxial Active style Pro device used in the Japanese studies may measure significantly less SB than the ActiGraph with the commonly used uniaxial cut-point of <100 counts per minute for SB (Yano et al., 2019), the lower estimates maybe have resulted from device differences.

Even though the majority of studies used ActiGraph devices, results from different ActiGraph models may not be comparable (Cain et al., 2013). Other variations in processing methods, such as non-wear algorithm length and choice of cut-points to define SB, will also affect sedentary time estimates (Mailey et al., 2014, Gorman et al., 2014). Included studies using uniaxial ActiGraph data all used a cut-point of <100 counts per minute for SB. While this cut-point is commonly used, evidence suggests that a lower cut-point is more appropriate when using the ActiGraph with older adults (Aguilar-Farias et al., 2014, Koster et al., 2016). The low-level light physical activities commonly seen in older adults may get counted as SB when using the cut-point of <100 counts per minute, thereby overestimating sedentary time (Koster et al., 2016). Because this cut-point was also used in previous studies with *younger* older adults, we can still conclude the oldest old age group is more sedentary than those age 60–80.

This review identified that age, gender, race/ethnicity, social disadvantage, and cognitive function may be associated with SB in adults aged 80 and older. However, except for gender, these factors were significant in only one study each, which will require additional studies to confirm them. In a previous review, additional factors were associated with SB in adults age 65 and older, including multiple personal factors (obesity, health status, retirement), environmental housing and neighborhood factors, and interpersonal factors related to living situations (Chastin et al., 2015). The small number of factors associated with SB in the current review indicates that the factors associated with SB have been inadequately studied among the oldest old.

Due to their cross-sectional design, the quality of the majority of included studies was rated as *fair*. The cross-sectional design presents a risk of bias for identifying factors that influence SB because it is difficult to assess the direction of causality. However, because articles with representative and larger samples were included, the cross-sectional design does not present a risk of bias for the first aim of this review, which is identifying the volume of SB in the targeted age group. Device-measured SB reduced the risk of bias in all studies, though the differences in measurement methods between studies presented challenges for synthesizing results.

Limitations and significant gaps in the science included less than optimal SB measures, infrequent analysis of factors influencing SB, and the use of activPAL, the most valid measure of SB (Kozey-Keadle et al., 2011), in only two unique studies. Also, uniaxial accelerometer cut-points for SB may not have been appropriate. Additionally, we found no evidence of modifiable risk factors for SB, factors that could be targeted in an intervention to reduce SB in the oldest old. A limitation of this review is that we only included articles published in English. Exclusion of articles in other languages could mean we are missing populations with potentially different patterns of SB.

This review has several important implications. The weighted mean of 10.6 sedentary hours/waking day highlights the magnitude of the SB problem and could be utilized to educate the oldest old adults on the potential for decreasing sedentary time. Although the factors associated with SB in this review need further verification, they provide preliminary evidence that certain subgroups of the oldest old may be at higher risk for elevated SB. This review of SB in community-dwelling oldest old will allow for comparison with older adults residing in residential care such as assisted living, most of whom are in this oldest old age group (National Center for Health Statistics, 2019).

In conclusion, this review found that adults age 80 and older are highly sedentary with a mean of 10.6 sedentary hours during the waking day. It is important to acknowledge that SB estimates were influenced by measurement methodology. Meta-analytic subgroup analyses revealed that older age and male gender may be related to increased sedentary time. Older age, male gender, non-Hispanic white race/ethnicity, social disadvantage, and cognitive function (in men) were the only factors found in individual studies to be associated with increased SB in this age group. These results highlights the need for future research to identify additional factors associated with SB.

Thank you to Kate Saylor for providing support as a health sciences informationist. Funding: K. W. was supported through NIH/NINR T32NR016914 when this review began and is now supported by NIH/NINR F31NR018784.

## CRediT authorship contribution statement

**Katelyn E. Webster:** Conceptualization, Formal analysis, Investigation, Writing - original draft, Visualization. **Weijiao Zhou:** Investigation, Validation, Writing - review & editing. **Nancy A. Gallagher:** Investigation, Validation, Writing - review & editing. **Ellen M. Lavoie Smith:** Writing - review & editing. **Neha P. Gothe:** Writing - review & editing. **Robert Ploutz-Snyder:** Formal analysis, Writing - review & editing. **Natalie Colabianchi:** Writing - review & editing. **Janet L. Larson:** Conceptualization, Investigation, Validation, Writing - review & editing, Supervision.

## Declaration of Competing Interest

The authors declare that they have no known competing financial interests or personal relationships that could have appeared to influence the work reported in this paper.
