# Supplementary material for: Device-measured sedentary behavior in oldest old adults: A systematic review and meta-analysis
Source: Prev Med Rep. 2021 May 18;23:101405. doi: 10.1016/j.pmedr.2021.101405 (PMC8181193; doi:10.1016/j.pmedr.2021.101405)
Supplement: Supplementary data 1 [file mmc1.docx]

Appendix Table 1. Objective measures of sedentary behavior: devices and data processing.

| Article Author (Year Published) | Device Used | Cut-Point or Determination of SB | Non-Wear Algorithm^a^ | Device Wear Period | Minimum Wear Requirements to be Included in Analysis | Mean Wear Time^b^ |
| --- | --- | --- | --- | --- | --- | --- |
| Arnardottir et al. (2012) | ActiGraph GT3X | <100 cpm | - | Waking hours for 7 days | 4 days with 10 hrs of wear time | Women 80-84 yrs: 13.4 hrs/day  Women ≥85 yrs: 12.9 hrs/day  Men 80-84 yrs: 13.6 hrs/day  Men ≥85 yrs: 13.6 hrs/day |
| Berkemeyer et al. (2016)^c^ | ActiGraph GT1M | <100 cpm | 90 min. | Waking hours for 7 days | 4 days with 10 hrs of wear time | 14.5 hrs/day  (all subjects 49-91 years) |
| Chastin et al. (2014) | ActiGraph AM-7164 | <100 cpm | 60 min. | Waking hours for 7 days | 5 days with 10 hrs of wear time (at least 1 weekend day) | - |
| Chen et al. (2015) | Active Style Pro HJA-350IT | ≤1.5 METS | 60 min. | Waking hours for 7 days | 4 days with 10 hrs of wear time | 13.8 hrs/day (all subjects ≥65 years) |
| Cukic et al. (2018) | activPAL3c | Thigh position | - | 24 hours for 7 days^d^ | 7 full days | - |
| Davis et al. (2011) | ActiGraph GT1M | <100 cpm | 100 min. | Waking hours for 7 days | 5 days with 10 hrs of wear time | 80-84 yrs: 13.9 hrs/day  ≥85 yrs: 14.1 hrs/day |
| Dunlop et al. (2015) | ActiGraph AM-7164 | <100 cpm | 60 min. | Waking hours for 7 days | 4 days with 10 hrs of wear time | 13.8 hrs/day^e^ |
| Evenson et al. (2012) | ActiGraph AM-7164 | <100 cpm | 60 min. | Waking hours for 7 days | 3 compliant days | - |
| Evenson et al. (2014) | NYC sample: ActiGraph GT1M and GT3X  NHANES sample: AM-7164 | <100 cpm | 60 min. | Waking hours for 7 days | 4 days with 10 hrs of wear time | NYC sample: 13.6 hrs/day (all subjects ≥60 yrs)  NHANES sample:14.1 hrs/day (all subjects ≥60 yrs) |
| Hooker et al. (2016) | Actical | <50 cpm | 150 min. | Waking hours for 7 days | 4 days with 10 hrs of wear time | 14.9 hrs/day^e^ |

Appendix Table 1 (continued)

| Jefferis et al. (2015) | ActiGraph GT3X | <100 cpm | 90 min. | Waking hours for 7 days | 3 days with 10 hrs of wear time | 14.1 hrs/day^e^ |
| --- | --- | --- | --- | --- | --- | --- |
| Lohn-Seiler et al. (2014) | ActiGraph GT1M | <100 cpm | 60 min. | Waking hours for 7 days | 4 days with 10 hrs of wear time | 14.0 hrs/day (all subjects 65-85 years) |
| Okely et al. (2019) | activPAL3c | Thigh position | - | 24 hours for 7 days^d^ | 7 full days | - |
| Rosenberg et al. (2020) | ActiGraph wGT3X+  ActivPAL micro | ≤18 vector magnitude counts/15 seconds  Thigh position | 90 min. (ActiGraph) | 24 hours for 7 days^d^ | 4 days with 10 hrs of wear time | Not given, but analyses adjusted for wear time |
| Ryan et al. (2019) | GENEActiv Original | Seated/reclined position with <0.057 Residual G (<1.5 METs) | - | 24 hours for 7 days | 6 days of 24 hr data | - |
| Sagelv et al. (2019) | ActiGraph wGT3X-BT | Vertical  <100 cpm  Vector Magnitude  <150 cpm | 20 min. | 24 hours for 8 days | 4 days with 10 hrs of wear time | ≥80 yrs: 16.2 hrs/day |
| Santos et al. (2018) | ActiGraph GT1M | <100 cpm | 60 min. | Waking hours for 4 days | 3 days with 10 hrs of wear time (including 1 weekend day) | Women 80-84 yrs: 13.6 hrs/day  women ≥85 yrs: 13.5 hrs/day  Men 80-84 yrs: 13.3 hrs/day  Men ≥85 yrs: 13.2 hrs/day |
| Shaw, Cukic, Deary, Gale, Chastin, Dall, Dontje et al. (2017) | activPAL3c | Thigh position | - | 24 hours for 7 days^d^ | 7 full days | - |

Appendix Table 1 (continued)

| Shaw, Cukic, Deary, Gale, Chastin, Dall, Skelton et al. (2017) | activPAL3c | Thigh position | - | 24 hours for 7 days^d^ | 7 full days | - |
| --- | --- | --- | --- | --- | --- | --- |
| Suzuki et al. (2020) | ActiGraph GT3X | <100 cpm | 60 min. | 24 hours for 7 days | 4 days with 10 hrs of wear time | Men: 18.5 hrs/day  Women: 18.4 hrs/day |
| Yonemoto et al. (2019) | Style pro HJA 350-IT | ≤1.5 METs | 60 min. | Waking hours for 7 days | 4 days with 10 hrs of wear time | ≥80 measured in 2009: 13.0 hrs/day  ≥80 measured in 2012: 13.2 hrs/day |
| Note: A hyphen indicates non-wear algorithm was not used/not reported or that mean wear time was not reported.  ^a^ Non-wear algorithm is the minimum length of little or no activity required for a period of time to be considered non-wear time (device was likely removed).  ^b^ Mean wear times reported apply to the same age group as the sedentary time results in Table 2 in the main text unless otherwise specified (wear time specific to the age category included in this review was not always reported).  ^c^ We only report on the EPIC-Norfolk dataset from this article. NHANES data is included in the article also, but is only reported graphically.  ^d^ Subjects kept a sleep diary that was used to isolate sedentary behavior during waking hours.  ^e^ Wear time not explicitly reported, but was calculated based on reported sedentary hrs/day and % of wear time in sedentary behavior. | | | | | | |
